# From inflammatory bowel disease to colorectal cancer: what’s the role of miRNAs?

**DOI:** 10.1186/s12935-022-02557-3

**Published:** 2022-04-11

**Authors:** Mostafa Vaghari-Tabari, Niloufar Targhazeh, Soheila Moein, Durdi Qujeq, Forough Alemi, Maryam Majidina, Simin Younesi, Zatollah Asemi, Bahman Yousefi

**Affiliations:** 1 Student Research Committee, Tabriz University of Medical Sciences, Tabriz, Iran; 2 Department of Clinical Biochemistry and Laboratory Medicine, Faculty of Medicine, Tabriz University of Medical Sciences, Tabriz, Iran; 3 Medicinal Plants Processing Research Center, Shiraz University of Medical Sciences, Shiraz, Iran; 4 Department of Biochemistry, Faculty of Medicine, Hormozgan University of Medical Sciences, Bandar Abbas, Iran; 5 Cellular and Molecular Biology Research Center (CMBRC), Health Research Institute, Babol University of Medical Sciences, Babol, Iran; 6 Department of Clinical Biochemistry, Babol University of Medical Sciences, Babol, Iran; 7 Solid Tumor Research Center, Urmia University of Medical Sciences, Urmia, Iran; 8 Schoole of Health and Biomedical Sciences, RMIT University, Melborne, VIC Australia; 9 Research Center for Biochemistry and Nutrition in Metabolic Diseases, Kashan University of Medical Sciences, Kashan, Iran; 10 Molecular Medicine Research Center, Tabriz University of Medical Sciences, Tabriz, Iran

**Keywords:** Inflammatory bowel disease, Colorectal carcinoma, miRNAs, Ulcerative colitis, Targeted therapy

## Abstract

Inflammatory Bowel Disease (IBD) is a chronic inflammatory disease with relapse and remission periods. Ulcerative colitis and Crohn’s disease are two major forms of the disease. IBD imposes a lot of sufferings on the patient and has many consequences; however, the most important is the increased risk of colorectal cancer, especially in patients with Ulcerative colitis. This risk is increased with increasing the duration of disease, thus preventing the progression of IBD to cancer is very important. Therefore, it is necessary to know the details of events contributed to the progression of IBD to cancer. In recent years, the importance of miRNAs as small molecules with 20–22 nucleotides has been recognized in pathophysiology of many diseases, in which IBD and colorectal cancer have not been excluded. As a result, the effectiveness of these small molecules as therapeutic target is hopefully confirmed. This paper has reviewed the related studies and findings about the role of miRNAs in the course of events that promote the progression of IBD to colorectal carcinoma, as well as a review about the effectiveness of some of these miRNAs as therapeutic targets.

## Introduction

IBD is a general term mainly referred to two categories of chronic inflammatory disorders of gastro-intestinal tract including Crohn's disease and Ulcerative colitis [1]. Crohn’s disease and Ulcerative colitis have very similar pathogenesis, and both can cause chronic bowel inflammation. IBD is developed in genetically predisposed individuals due to improper immune response to intestinal bacteria [2]. It’s safe to say that IBD is one of the most complex human diseases and several factors are involved in the pathogenesis of IBD. Recent reports also indicate an increase in the prevalence of this disease, especially in Europe [3]. Despite many studies, the exact mechanisms of IBD pathogenesis are still unclear. Diagnosis and monitoring of the disease has always been a serious challenge, thus many studies have focused on identifying appropriate biomarkers for diagnosis and monitoring patients [4–7]. Besides, Ulcerative colitis and Crohn’s disease are also very similar in clinical, endoscopic and histological features, and it is difficult to differentiate between the two diseases and is a serious challenge for gastroenterologists. Therefore, some studies have focused on finding helpful indicators for differential diagnosis. For example, it seems that ANCA positivity is more prevalent in Ulcerative colitis but ASCA positivity is more frequent in Crohn’s disease. Crohn’s disease has been shown to affect any area of the GI tract, while Ulcerative colitis affects the colon and rectum. Inflammatory lesions in Ulcerative colitis are limited to mucus and continuous, while in Crohn's disease it is transmoral and discontinuous. Granuloma can also be a diagnostic clue to Crohn’s disease and is very rare in Ulcerative colitis. Differential diagnosis can be useful in more effective treatment. For example, antibiotic therapy is recommended in the treatment of Crohn's disease, or 5-ASA appears to be more effective in maintaining remission in Ulcerative colitis than Crohn's disease. Also, finding appropriate and novel treatment approaches is another part of IBD-related research [8, 9]. On the other hand, the risk of colorectal cancer in patients with IBD is a serious problem. The results of a meta-analysis showed that, after 10 years of diagnosing Ulcerative colitis, the risk of colorectal cancer is 0.02%, after 20 years is 4.8% and after 30 years is 13.9% [3]. Another meta-analysis has shown that these values in patients with Crohn’s disease were 2.9%, 5.6% and 8.3%, respectively [10]. Therefore, the risk of colorectal cancer is increased with the increase in duration of IBD. Accordingly, this study has also focused on the identifying mechanisms that are involved in the progression of bowel chronic inflammation to colorectal cancer.

**Fig. 1:**
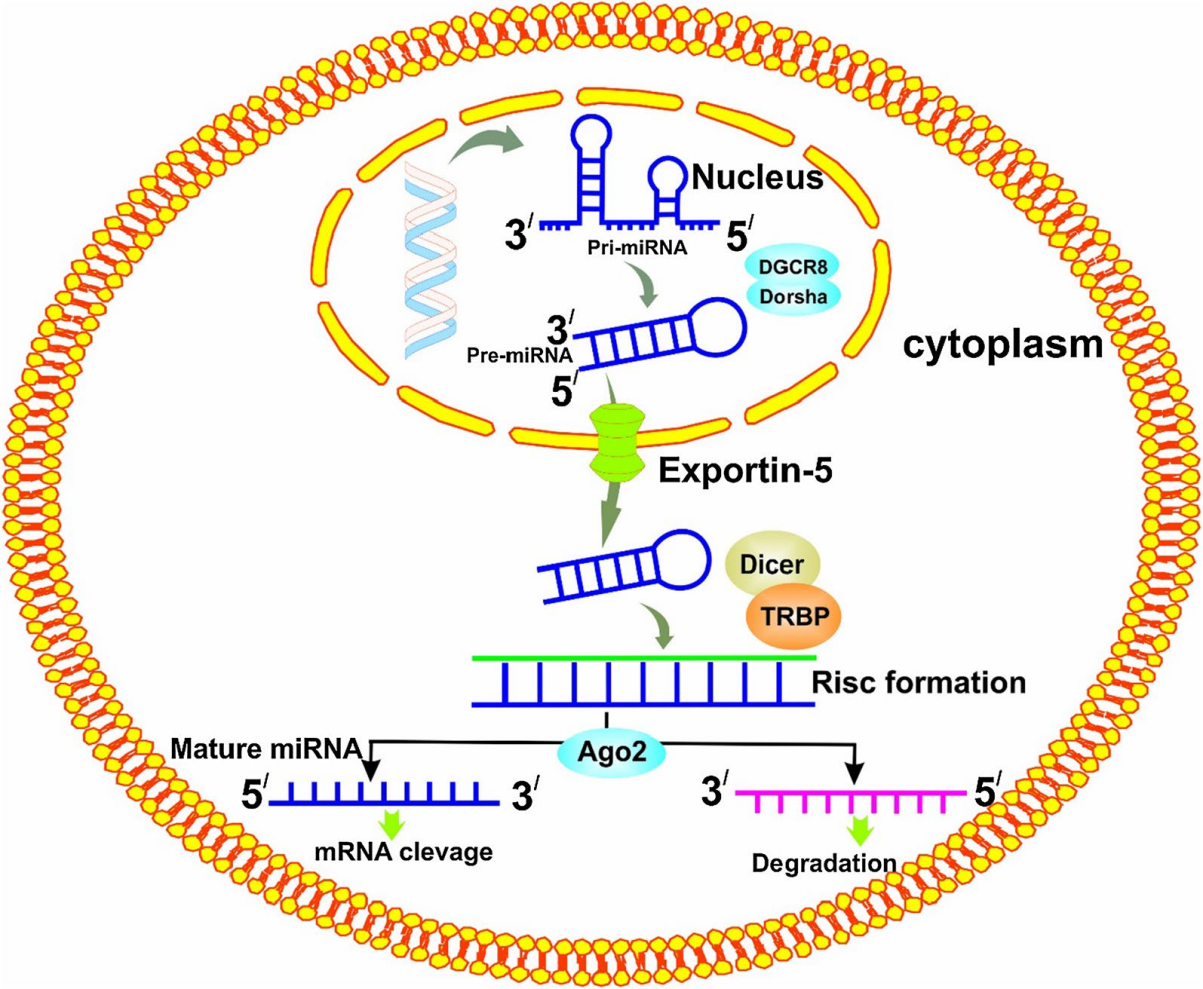
Schematic diagram representing multiple steps in biogenesis of microRNA

In this regard, miRNAs are considered as molecules that are important in all of the mentioned cases. miRNAs are small RNA molecules with 20 to 22 nucleotides. The gene encoding miRNAs is transcribed by RNA Pol II enzyme. The primary transcription of miRNAs (called Pri-miRNA) is converted to Pre-miRNA inside the nucleus. Pre-miRNA goes to the cytoplasm and after a series of interactions, ultimately becomes a mature miRNA that binds to the target mRNA with the help of proteins called argonaute, which causes mRNA instability or translating suppression (Fig. 1) [11]. In recent years, the importance of these small molecules has been recognized in the molecular mechanisms of many human diseases and considered as a therapeutic target for many human diseases including IBD and colorectal cancer [12, 13]. Thereafter, this review paper has tried to review the latest findings on the role of miRNAs in these events in addition to reviewing the events involved in the progression of IBD to colorectal cancer.

## Colitis associated cancer

Colorectal cancer (CRC) is one of the most common malignancies. As reported by the United States Cancer Society in 2017, colorectal cancer is the third most common cancer in men and women in United States [14]. Colorectal cancer is divided into three types of familial, sporadic, and inflammatory related with important differences. In general, familial type mutations are inherited in *APC* gene or genes involved in mismatch repair, and other mutations in tumor suppressor genes such as *TP53* or proto-oncogene like *KRAS* occur gradually. However, in sporadic type, genetic changes and interactions that lead to tumorigenesis usually occur with aging. Inflammatory related type of colorectal cancer is called colitis associated cancer (CAC) or IBD-associated cancer in literature. In this type of colorectal cancer, chronic inflammation is main cause of tumorigenesis and has significant differences with other types of colorectal cancer. In CAC mutation in *APC* gene is not very common and its occurring is in latter stages. Mutation in *TP53* tumor suppressant gene occurs in early stages, while in sporadic and familial types, mutation occurs in *TP53* gene and in last stages [15–17]. About 15% of death’s causes belong to CAC among IBD patients with a weaker prognosis compared to other cases [18]. The risk of CAC has a direct relation to the duration of IBD, as well as the extent of inflamed areas [3, 19].

A series of events that occur in IBD including the penetration of immune cells into lamina propria, various cytokine secretions, oxidative stress, and etc., have effects on many genetic factors and cellular signaling pathways, which lead a normal epithelial cell to Dysplasia and metastatic adenocarcinoma. The course of these events from bowel chronic inflammation to cancer and the role of miRNAs in these events will be discussed.

## From IBD to cancer

As mentioned above, IBD is an umbrella term that refers to Ulcerative colitis and Crohn's disease. Because both Crohn’s disease and Ulcerative colitis can cause chronic inflammation in the colon tissue and this chronic inflammation is associated with an increased risk of colorectal cancer, this review article uses the general term (IBD) to discuss the relationship between bowel chronic inflammation and colorectal cancer. The progression of IBD to colorectal cancer is another confirmation of the role of chronic inflammation in cancer development. Chronic inflammation in long term leads to epithelial cells moving toward Dysplasia and ultimately adenocarcinoma. In this long course, several factors such as DNA damage [20] and mutation in *TP53* tumor suppressant gene, immune cells, and cytokines produced by them, signaling pathways such as NFκB, STAT3, AKT/PI3K [21], and WNT/β-Catenin [22] are involved and miRNAs are also associated with all of these factors in some way. It seems that targeting some of them can be useful in stopping this dangerous course or more effective treatment of colorectal cancer. Below we will cover all these events from IBD to colorectal cancer and will review the latest findings on the role of miRNAs**.**

### IBD, immune cells and CAC: a brief overview

Immune system plays a central role in the pathogenesis of IBD. In fact, this is an inappropriate response of the immune system to some intestinal microorganisms, which, in addition to the genetic predisposition, leads to the onset of IBD. Inflammation has no harmful nature and is an immune response to infection or damage with a beneficial nature led to the removal of aggressive agents, repairing structures and restoring tissue function. Acute phase of inflammation is associated with the rapid flow of immune cells, especially neutrophils and inflammatory macrophages to the affected tissue. Inflammatory macrophages have effect on the performance of tissue macrophages. In addition, several cytokines are produced by neutrophils and macrophages that play a very important role in immune response. These interactions cause common symptoms of inflammation such as heat, pain and swelling. When the harmful stimulus disappears, the inflammatory reaction is subsided, and the immune cells are returned to the phenotype and the number before the inflammatory reaction. In fact, acute inflammation is resolved and the tissue is restored, but if there is a disturbance in the resolving of acute inflammation, chronic inflammation will be developed resulted an increased tissue damage just as in IBD [23]. In intestine, a mucosal barrier prevents microbes from penetrating into lamina propria. The stability of this mucosal barrier depends on the existence of tight junctions between epithelial cells. In IBD, these tight junctions are loosened, so the mucosal barrier is weakened and the bacterial penetration increased to lamina propria which is followed by neutrophils, then macrophages enter to the lamina propria to eliminate these bacteria and an inflammatory reaction begins. In IBD, CD4^+^ T cells and innate immune cells such as neutrophils and macrophages interact closely with each other. Thus the inflammatory signaling pathways such as NFκB and STAT3, as well as inflammatory cytokines, mediate these interactions in a way that Innate immune cells secrete cytokines such as TNF-α, IL-6, IL-1, IL-12, IL-21, IL-22 and IL-23 resulted an increasing of pro-inflammatory CD4^+^ T cells recruitment to lamina propria. These cells also secrete cytokines that increase the number of innate immune cells which is resulted in the continued cycle of inflammatory response and chronic inflammation [13, 24, 25]. Some of these cytokines can directly affect the epithelial cells and stimulate signaling pathways which are involved in tumorigenesis.

T cells, based on their glycoprotein co-receptor that they have in their surface, are divided into two major CD4^+^ and CD8^+^ groups. CD4^+^ T cells play an important role in the pathogenesis of IBD and CAC and divided into two groups under the names of effector cells and regulatory cells [13]. Th1, Th2 and Th17 are three main categories of T-effector cells and secrete very important cytokines. Interferon γ is the most important cytokine produced by Th1 cells, while Th2 cells secreting IL-4 significantly. IL-17, IL-22 and IL-21 are also secreted by Th17 cells. Regulatory T cells play a role in regulating the activity of T-effector cells and immune tolerance, and secrete cytokines such as TGF-β usually play anti-inflammatory activity role. CD8^+^ T cells which are known as killer cells are closely interacting with T-effector cells. In fact, the function of CD8^+^ T cells that is very important in the fight against cancer cells is dependent on receiving signals from CD4^+^ T cells [13]. Th1 and Th2 cells appear to have opposing roles in CAC so that Th1 cells play a protective role; however, Th2 cells interfere with tumor stimulation [26, 27]. Despite the high similarities in the immune-pathogenesis of Crohn’s disease and Ulcerative colitis, there are some differences in the adaptive immune response between the two diseases. Th2 has been shown to be more involved in the pathogenesis of Ulcerative colitis, but, Th1 and Th17 are more involved in the pathogenesis of Crohn’s disease [13, 28]. Some of the phenotypic differences between Crohn’s disease and Ulcerative colitis mentioned in the introduction may be related to these differences in the adaptive immune response. For example, transmural inflammation in Crohn’s disease may be due to the predominant immune response of Th1 and Th17 cells, whereas mucosal inflammation in Ulcerative colitis is probably due to a more pronounced Th2 immune response [28]. Granuloma formation in Crohn's disease also appears to be associated with Th1 immune response and interferon γ [29, 30]. Granuloma formation begins following an inflammatory stimulus. As mentioned above, macrophages are recruited to the site of inflammation, as an important part of the innate immune response. Due to the events mentioned above and the chronic inflammation in Crohn's disease, this recruitment can occur continuously. Macrophages, in turn, by secreting cytokines such as TNF-α, IL-1, IL-6 and IL-12 can enhance leukocyte infiltration and T cell activation. Th1 cells can also further enhance the recruitment of macrophages to the area of inflammation by secreting chemokines and cytokines such as TNF-α, interferon γ, and IL-6. In a study of colon tissue granulomas of patients with Crohn's disease significant immunoreactivity was reported for IL-12 and interferon γ.

The secretion of interferon γ by Th1 cells is also involved in shifting the polarization of macrophages to the pro-inflammatory phenotype (M1), which will be discussed below [30–32]. Continuation of successive cycles of inflammation and secretion of TNF-α and interferon γ by macrophages and Th1 cells leads to further maturation of macrophages [33]. This maturation of macrophages causes the formation of structures called epithelioid cells that are similar to epithelial cells. These epithelioid cells fuse together to form multinucleated giant cells, and eventually a structure called a granuloma can form [31]. In fact, granuloma is an organized structure consisting of activated macrophages aggregate (epithelioid and multinucleated giant cells), which are surrounded by lymphocytes [34]. As mentioned in the introduction, granuloma can be an endoscopic feature of Crohn’s disease that can help to distinguish Crohn's disease from Ulcerative colitis. Neutrophils and macrophages role are also inevitable in the progression of IBD to cancer. Neutrophils, in addition to the role played by inflammatory cytokines, play an important role in increasing the risk of colorectal cancer in patients with IBD by production of free radicals and carcinogenic compounds such as N-nitroso [35, 36]. Macrophages also play an important role in IBD and cancer. These cells are divided into two types of inflammatory M1 and anti-inflammatory M2. Inflammatory macrophages mainly inhibit cell proliferation, while M2 macrophages play a role in stimulating cell proliferation. Inflammatory macrophages release high levels of free radicals as well as IL-6, which play an important role in tumorigenesis. M2 macrophages play a role in neutralizing the destructive effects of these compounds, as well as repairing tissue damage. In fact, there is a balance between these two macrophages. Studies have shown that this balance is changed in progress of Dysplasia toward colorectal carcinoma in favor of M2 macrophages [13, 37]. In fact, M1 macrophages seem to play a role in cellular damage and initiate the process of tumorigenesis, while these are M2 macrophages that play a major role in progressing towards colorectal carcinoma. Another group of immune cells that appear to be involved in the pathogenesis of IBD are innate lymphoid cells (ILCs). These cells are originated from common lymphoid progenitors (CLPs) and divided into three groups, which are named from 1 to 3. NK cells and ILC1s are in group 1, ILC2s are in group 2 and ILC3s are in group 3 of these cells [38]. The number of NK cells in the lamina propria of patients with IBD (both Ulcerative colitis and Crohn's disease) appears to be increased [39].

These cells may be involved in the pathogenesis of IBD by secreting interferon γ and enhancing the differentiation of Th1 cells from naive CD4^+^ T cells. In addition, excessive amount of interferon γ has destructive effects on the tight junctions of the intestinal mucosal barrier [40], which is an important event in strengthening chronic inflammation in IBD. However, some studies in animal models of colitis have shown that NK cells, through NKG2A inhibitory receptors and direct cell–cell contact, attenuate the pro-inflammatory functions of neutrophils, including cytokine and ROS secretion, and have protective effects against colitis [41]. ILC1 cells have the ability to secrete interferon γ and express T-bet, which is a transcription factor. These cells can be developed from ILC3 cells [RORγt (+) ILC3] under the effect of IL-12. It seems that the frequency of ILC1 cells in the inflamed intestine of patients with Crohn’s disease increases [42]. Increased frequency of ILC1 in inflamed intestinal tissue of patients with established Ulcerative colitis has also been reported [43]. These findings suggest the role of ILC1 in chronic bowel inflammation. However, the exact mechanism of function of these cells in the pathogenesis of IBD is not yet clear and needs to be further studied. Increased ILC2 cell populations have also been reported in inflamed tissues of patients with newly diagnosed Ulcerative colitis, and patients with established Crohn's disease and Ulcerative colitis [43]. These cells have the ability to secrete IL-13 and IL-5, and in fact provide a primary source of Th2 cytokines [40]. ILC2 cells appear to be involved in maintaining the integrity of the intestinal mucosal barrier In addition, IL-13 derived from these cells may be involved in enhancing the differentiation of intestinal stem cells into Goblet and Turf cells and repairing intestinal damage [44]. IL-33, which can be released from damaged epithelial cells [45], appears to have an effect on ILC2 cells and this effect is involved in the pathogenesis of IBD, but contradictory results have been published in this regard. One study found that IL-33 deficiency impaired the differentiation of ILC2 and Th17 cells, and reduced levels of cytokines such as IL-6 and IL-1, which protected mice against DSS-induced colitis. The results of this study also showed that exogenous IL-33 causes exacerbation of colitis [46]. However, the results of another study showed that IL-33 had a protective effect against DSS-induced colitis by enhancing the expansion of ILC2 and Treg cells [47]. Therefore, further studies are needed to determine the exact mechanism of action of ILC2 cells in the pathogenesis of IBD. Besides, given that IL-33 appears to be involved in the progression of colorectal adenoma to colorectal cancer [48], it is necessary to study the role of ILC2 cells in the development of CAC.

ILC3 cells are another group of innate lymphoid cells that appear to be involved in the pathogenesis of IBD and CAC. Continuous expression of RORγt and aryl hydrocarbon receptor (AHR) is necessary for the differentiation and survival of these cells [38].Lymphoid tissue inducer (LTi) cells, NKp44^+^ ILC3s and NKp44^−^ ILC3s are subgroups of ILC3 cells. The majority of the populations of ILC cells in the intestinal lamina propria are NKp44^+^ ILC3s cells [40]. Interestingly, one study showed that the frequency of these cells in the inflamed tissue of IBD patients (both Ulcerative colitis and Crohn’s disease) was reduced [43]. These cells have the ability to produce IL-22 [40]. This cytokine has protective effects on IBD and can enhance the integrity of the intestinal mucosal barrier and induce antimicrobial compounds [49]. However, IL-22 can enhance STAT3 signaling in epithelial cells, increase proliferation, and play a role in colorectal cancer development [50]. NKp44^+^ ILC3s present in the inflamed intestinal tissue of patients with Crohn's disease appear to secrete less IL-22 but are capable of secreting interferon γ. NKp44^+^ ILC3s present in the inflamed intestinal tissue of patients with Crohn's disease appear to secrete less IL-22 but are capable of secreting interferon γ. These cells also enhance the accumulation of inflammatory monocytes by secreting GM-CSF, leading to promotion of intestinal inflammation [40]. Therefore, these cells seem to be involved in the pathogenesis of IBD and CAC, but their exact role is not yet clear and requires further study. Another group of immune cells that appear to be involved in the pathogenesis of IBD are NKT cells. The development of these cells depends on the thymus and most of them branch from CD4^+^ CD8^+^ double-positive (DP) thymocytes. In fact, majority of NKT cells branch out from the conventional development of T cells in the CD4^+^ CD8^+^ DP step [51]. These cells express surface molecules of both T cells (such as TCR and CD3) and NK cells (such as NKG2D and CD161), are divided into two types based on differences in TCR [52]. The population of NKT type 1 cells in the intestinal tissue and peripheral blood of IBD patients (both Crohn's disease and Ulcerative colitis) appears to decrease, but a significant accumulation of NKT type 2 cells in the intestinal tissue of patients with Ulcerative colitis has been reported [53]. It seems that the pathogenic role of NKT type 2 cells in Ulcerative colitis is exerted by the secretion of IL-13 and this cytokine can cause apoptosis of intestinal epithelial cells and play a role in the destruction of intestinal mucosal barrier. NKT type 1 cells from the lamina propria of patients with Crohn's disease also have the ability to secrete pro-inflammatory cytokines, such as TNF-α,IFN-γ, and IL-13, and are involved in the destruction of the intestinal mucosal barrier [54, 55].

**Fig. 2:**
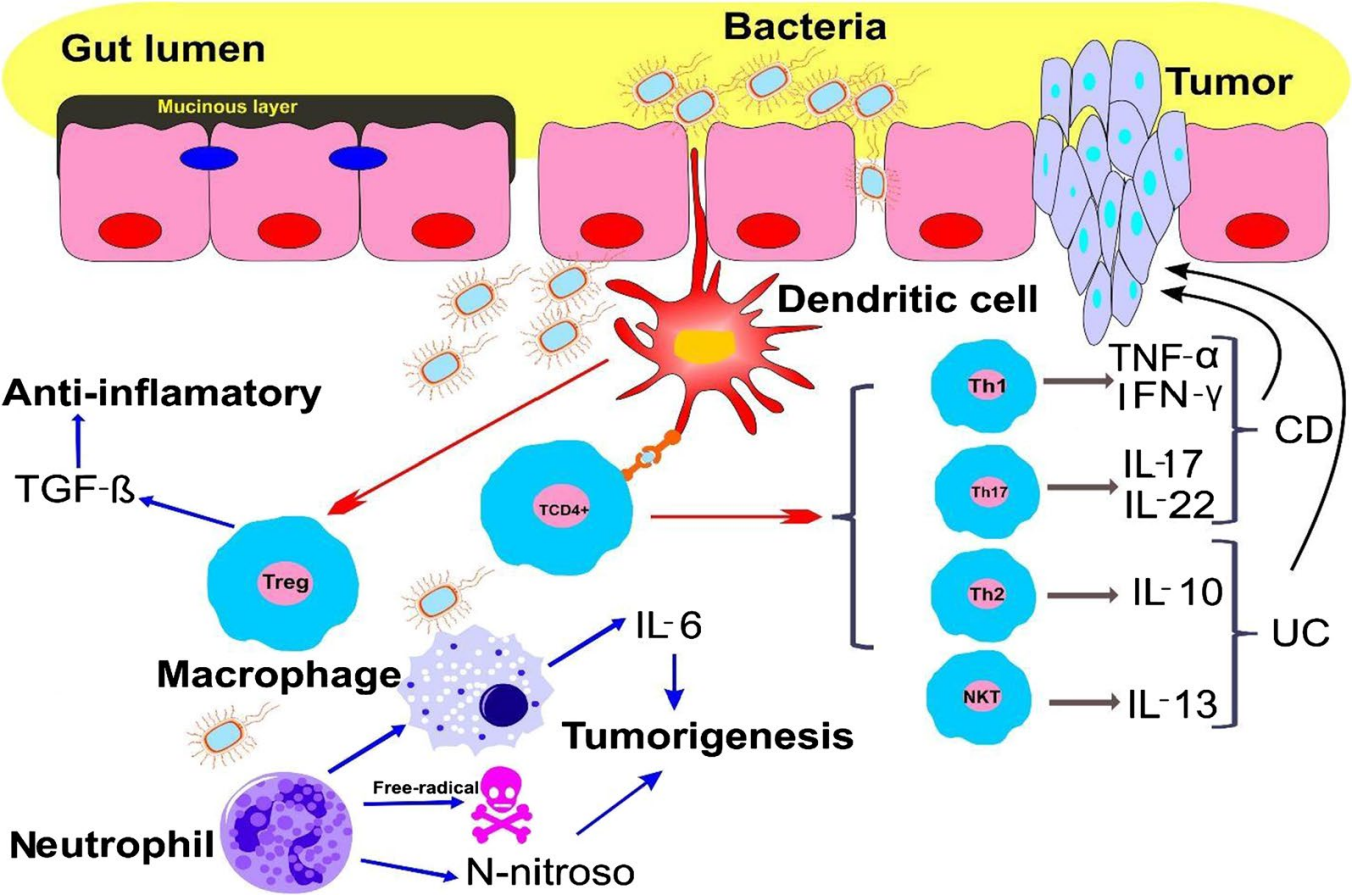
Schematic diagram represents the regulation of epithelial cells behavior by immune system in IBD and CAC

Some studies have also shown that NKT type 1 cells have protective effects against colitis in mice by secreting IL-9 [56].In overall, type1 NKT cells appear to have both a protective and a pathogenic role in IBD but type2 NKT cells may enhance intestinal inflammation [52]. All of immune cells and cytokines produced by them involved in bowel chronic inflammation have an important role in developing CAC. As previously mentioned, the risk of CAC induced colorectal cancer among IBD patients. In recent years, extensive studies have been conducted to find out how IBD increases the risk of colorectal cancer. These studies have shown that chronic inflammation (a major characteristic of IBD) has harmful effects on intestinal epithelial cells. Free radicals and inflammatory mediators can cause DNA damage and mutation in important genes such as *TP53* tumor suppressant gene. Additionally, chronic inflammation causes the epithelial cells to acquire the characteristics of stem cells which is called epithelial–mesenchymal transition (EMT) and eventually become a tumor cell [57]. Studies also provide significant information on cytokines produced by immune cells, key signaling pathways in developing CAC and also the role of miRNAs in all of these interactions (Fig. 2). Inflammation that is induced by microorganism leads to the removal of mucinous layer and penetration of bacteria in lamina propria and stimulate immune cell. The production of cytokine and inflammatory factors lead to the progression of IBD to colorectal cancer.

### miRNAs, oxidative stress and DNA damage

DNA damage and mutation can be the starting point for cancer development. Of course, cell has mechanisms to repair these injuries. If these mechanisms fail to resolve the damage, cell destroys itself with apoptosis process. However, if the apoptosis has not happened, some of these catastrophic mutations such as mutations in *TP53* tumor suppressant gene push the cell into malignancy. In IBD patients, beside antioxidant defense impairment, the presence of oxidative and nitrosative stress and the production of free radicals of oxygen and nitrogen in high amounts by neutrophils, macrophages and epithelial cells have been observed [58–61]. Free radicals not only damage DNA, but also appear to disrupt some repair mechanisms including mismatch repair [62]. Some inflammatory cytokines such as IL-6 and IL-22 also activate the signaling pathways of NFκB and STAT6 in epithelial cells.

These signaling pathways stimulate the expression of anti-apoptotic genes, so chronic inflammation not only causes DNA damage and impaired some restorative processes like mismatch repair, but also eliminates the cell from apoptosis, raising the risk of colorectal cancer in patients with IBD. Oxidative stress occurs when the balance between the production of free radicals and their clearing by antioxidant system is interrupted. In IBD, immune cells including macrophages and neutrophils, in addition to produce a high level of free radicals themselves by inducing enzymes such as NADPH oxidase and nitric oxide synthase (NOS) in colon cells could produce a high level of ROS and RNS [61]. The role of nitric oxide in pathogenesis of IBD and creation of DNA damage is very interesting. Nitric oxide can react with anion superoxide (a highly reactive free radical) and generates radical peroxynitrite (ONOO^−^) which is a very destructive oxidant factor in causing DNA fragmentation [61]. In addition, nitric oxide stimulates the production of TNF-α which is playing significant role in the pathogenesis of IBD and causing mutation and progressing to CAC. Accordingly, downregulation of nitric oxide synthase reduces the severity of colitis in mouse models [63, 64]. Furthermore, it has been shown that mesalazine (commonly used drug for IBD treatment) has antioxidant properties [65]. Some studies have also shown that some antioxidant compounds can be effective in protecting against DNA damage, as well as reducing colitis severity or even inhibiting the signaling pathway of WNT/β-Catenin, which is very important in colorectal cancer development and invasion [66–68]. Although the role of free radicals in IBD and the early stages of tumorigenesis is a destructive role, it seems that these highly reactive compounds are useful in preventing invasion and in colorectal cancer treatment [69, 70], while antioxidants may play a destructive role in the end stages. It seems that, superoxide dismutase (an antioxidant enzyme) has been involved in EMT and tumor cell invasion in colorectal cancer [71]. In addition, strengthening the antioxidant system seems to be one of the mechanisms of resistance to chemotherapy in colorectal cancer cells [12]. The relationship between miRNAs and oxidant and antioxidant compounds is very interesting topic addressed recently. For example, it has been shown that miR-212 by targeting superoxide dismutase mRNA neutralizes the role of this enzyme in invasion of tumor cells [71]. MiR-143 is also another miRNA whose effects have been shown to induce apoptosis and inhibit the proliferation of colorectal cancer cells [72].

**Fig. 3:**
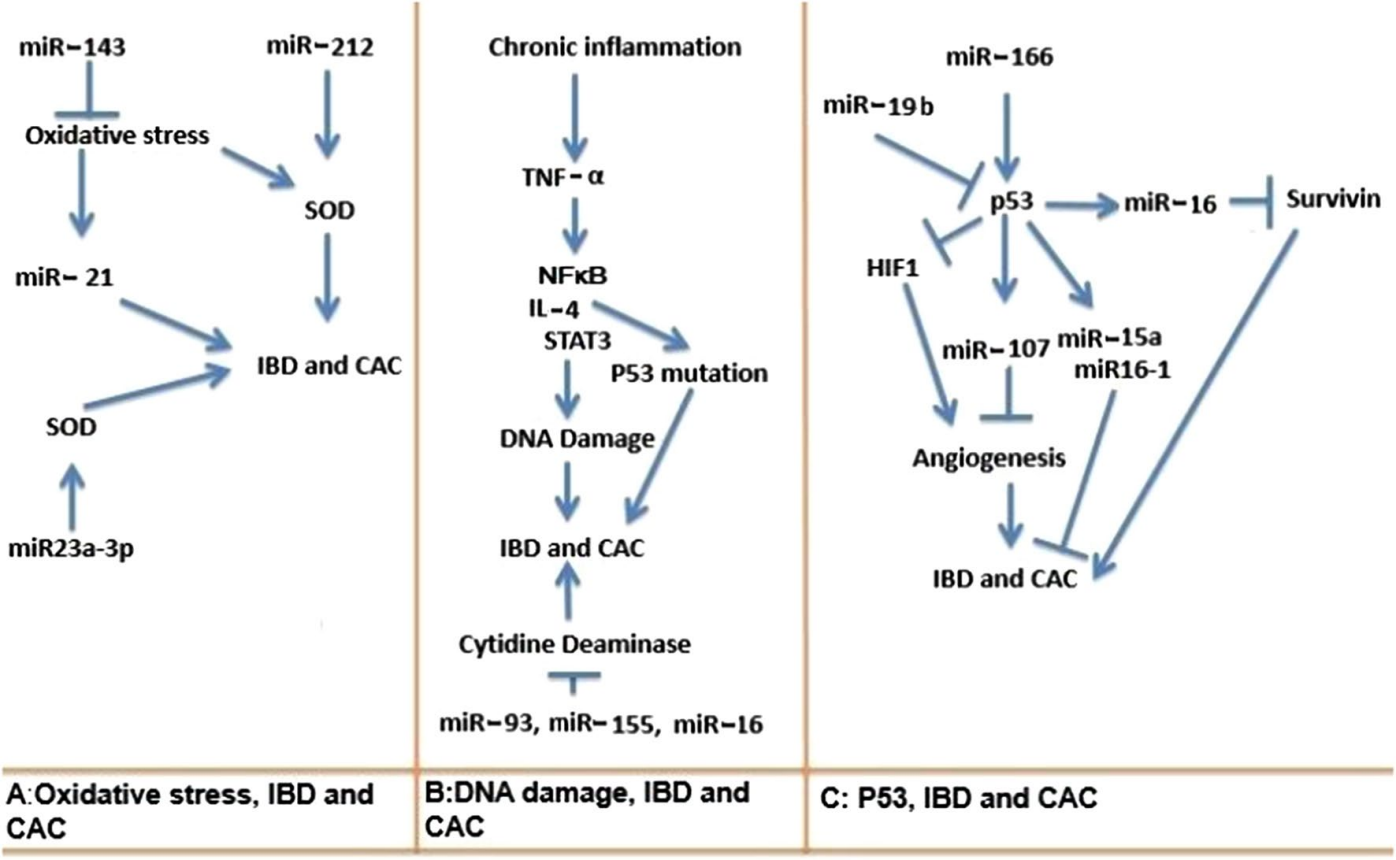
Crosstalk between inflammatory factors, miRNAs in oxidative stress, DNA damage and p53 functions during IBD and CAC

By exacerbating the oxidative stress, this miRNA reduces the resistance of colorectal cancer cells to treatment, thus it seems that the useful role of free radicals in colorectal cancer treatment appears to interact with miRNAs such as miR-143 [69] (Table 1). Adding that the harmful roles of free radicals as important factors in IBD and in the early stages of tumorigenesis are also likely to interact with miRNAs. For example, free radicals can activate miR-21 as important oncogenic microRNA and more interestingly this miRNA increases the production of free radicals by targeting antioxidant enzyme superoxide dismutase, suggesting a close relationship between this oncomiR and free radicals [73, 74]. MiR-21 also plays a very active role in the pathogenesis of IBD and is probably one of the miRNAs that play a very important role in colorectal cancer development in patients with IBD [13]. Some miRNAs such as miR-23a-3p play a protective role against oxidative stress. This miRNA increases the expression of superoxide dismutase enzyme, requiring more studies on IBD and colorectal cancer [75]. Identifying the mechanism of action and the use of these miRNAs or inhibiting miRNAs such as miR-21 may have a protective effect against oxidative stress and subsequent oxidative DNA damage in IBD patients and prevent the onset of tumorigenesis and progression towards colorectal adenocarcinoma. Chronic inflammation can also cause damage to DNA and mutation without free radical involvement. TNF-α increases the expression of activation induced cytidine deaminase (AID) in colon epithelial cells by activating NFκB and IL-4/STAT6 signaling pathways [76, 77]. This enzyme causes deamination and conversion of cytosine base to uracil in DNA structure, and it seems to play an important role in creating and development of CAC [77–79]. Also, activation of this enzyme in the following of inflammation reaction is associated with an increase in the mutation of *TP53* gene in colon cells [76]. Interestingly, it has been shown that in IL-10 (−/−) AID (−/−) mouse models, despite chronic inflammation, colon cancer does not occur and mutations in the *Tp53* gene is significantly lower [80]. According to these findings, AID inhibition can be an effective approach to prevent CAC in IBD patients, and this inhibition can be done by miRNAs such as miR-93, miR-155 and miR-16 [81, 82] (Fig. 3A and B).

**Table 1:** Summery of miRNAs roles in bowel inflammation and colorectal cancer

miRNAs	Target	Possible function/effect	References
miR-212	SOD	Decreasing the levels of MnSOD	[71]
		Attenuation EMT in colorectal cancer cells	
		Attenuation of invasiveness of colorectal cancer cells	
miR-143	SOD	Down-regulation of SOD	[69]
		Enhancement oxidative stress	
		Enhancement of chemo-sensitivity of colorectal cancer cells	
miR-150-5p	p53	Inhibiting p53	[227]
		Enhancement proliferation of colorectal cancer cells	
miR‑766	MDM4	Enhancement of p53/BAX signaling	[228]
		Enhancement of apoptosis in colorectal cancer cells	
miR-107	HIF-1β	Inhibiting HIF-1β expression in colorectal cancer cell	[85]
		Mediating p53 effects on hypoxic signaling	
		Attenuation of angiogenesis and tumor growth	
miR-124	DNMT3B	Attenuation of DNA methylation	[104]
miR-506	DNMT1	Inhibition of colorectal cancer progression	
miR-342	DNMT1	Inhibiting of proliferation and invasion of colorectal cancer cells	[105]
miR-143	DNMT3A	Attenuation colorectal cancer cells growth	[106]
miR-155	claudin1	Up-regulation of claudin1 in colorectal cancer cells	[127, 192]
	Jarid2	Enhancement of invasion and migration of colorectal cancer cells weakening intestinal mucosal barrier in IBD	
		Down-regulation of Jarid2induction of Th17 cells differentiation in mouse models of colitis	
		Enhancing IL-22 expression in mice models of colitis	
miR-19-a	TNF alpha-induced protein 3	Enhancing NFκB signaling	[130]
		Increasing the production of TNF-α	
		Promoting colitis and CAC in mouse models	
miR-21	PTEN	Enhancing proliferation and attenuating apoptosis in colorectal cancer cells	[141, 147, 229]
	PTEN	Enhancing NFκB signaling	
		Enhancing IL-6 production weakening of intestinal mucosal barrier in IBD	
		Increasing TNF-α and MIP-2 in mice colon	
miR-181b	PDCD4	Mediating the effects of IL-6/STAT3 signaling on PDCD4	[142]
		Down-regulation of PDCD4	
		Enhancing proliferation and attenuating apoptosis in colorectal cancer cells	
miR-34a	S-IL-6-R1	Attenuating IL-6 effect on epithelial cells	[148, 149]
		Attenuating IL-6/STAT3 signaling in colorectal cancer cells	
		Mediating p53 suppressing effects on invasion and migration in colorectal cancer cells	
miR-214	PTEN	Mediating IL-6 suppressing effects on PTEN in colorectal cancer cells	[150]
	PDZ and LIM domain 2	Enhancement of PI3K/AKT and NFκB signaling in colon tissue	
		Enhancement of inflammation in Ulcerative colitis	
		Promoting the progression of colitis toward CAC	
miR-139-5p	NFκB	Attenuation the expression of IL-6 and TNF-α in colorectal cancer cells	[153, 154]
		Suppressing colorectal cancer cells proliferation	
		Protection against colitis and colorectal cancer	
miR-200b	AKT2	Attenuating AKT expression in colon tissue	[156]
		Attenuating NF-κB/IL-6/STAT3 signaling	
		Down-regulation of TNFα	
		Attenuating inflammatory response in mice colon	
		Attenuating EMT	
miR-223	Claudin-8	Mediating IL-23 inhibitory effects on Claudin-8	[165]
		Weakening epithelial barrier of intestine	
		Enhancing intestinal inflammation	
miR-29	IL-23	Down-regulation of IL-23 in dendritic cells	[166]
		Attenuating colitis	
miR-193a-3p	IL-17RD	Down-regulation of IL-17RD	[172]
		Down-regulation of p-AKT	
		Reducing EGFR signaling in colorectal cancer cells	
		Attenuation colorectal cancer cells proliferation	
		Protection against CAC	
miR-124	STAT-3	Reducing IL-17 expression in Th17 cells	[174, 175]
		Attenuating the differentiation of Th17 cells	
		Protection against inflammation exacerbation in Ulcerative colitis	
		Attenuating CAC development in mice	
miR-106a	IL-10	Negatively regulating IL-10 expression	[215, 230]
		Attenuating Treg cells suppressive function	
		Stimulating intestinal inflammation in mice	
miR-27	Smad2	Down-regulating p-STAT3	[222]
	Sphingosine-1-phosphate phosphatase 1	Inhibiting colorectal cancer cells proliferation	
		Enhancing apoptosis in colorectal cancer cells	
miR-140-5p	Smad2	Decreasing Smad2 expression	[225]
		Decreasing colorectal cancer cells proliferation	
		Attenuating colorectal cancer cells invasion

### Inflammation, P53 mutation and miRNAs

*TP53* is the most important tumor suppressant gene in the body's cells, which encodes p53. In the presence of damage in DNA, p53 is phosphorylated and activated by ATM and ATR protein kinases. Activated p53 increases the P21 production in the cell, and by preventing phosphorylation of retinoblastoma protein activates P21, subsequently, stops the cell cycle in G1 phase so that repairing systems can repair DNA damage. In addition, P53 through increasing the transcription of apoptotic proteins plays a key role in induction of apoptosis [83], so mostly the mutation of genes of this tumor suppressor leads to cancer development. In many cancers including colorectal cancer, mutation occurs in *TP53* gene. As previously mentioned, contrary to sporadic type in CAC, mutation occurs in *TP53* gene in the first stages of Dysplasia. In fact, the early start of IBD progression towards Dysplasia is caused by the harmful effects of free radicals and inflammatory interactions on DNA. Subsequently, the impairment of repair and mutation processes in *TP53* gene along with chronic inflammation resulted in the continuous production of inflammatory cytokines and high activity of STAT3 and NFκB signaling pathways have anti-apoptosis properties running Dysplasia towards colorectal adenocarcinoma. Alternatively, more mutations in *TP53* gene of colon tissue with Ulcerative colitis compared to healthy people as another reason for high risk of colorectal cancer developing have been studied [84]. p53 seems to apply some of its anti-tumor effects by miRNAs. For example, p53 inhibits hypoxia-inducible factor 1 (HIF1) which is an angiogenesis stimulator transcription factor by stimulating the production of miR-107 and inhibiting angiogenesis (a vital process for cancer cells) [85] (Table 1). p53 also has an inhibitory effect on EMT via miR-15-a and miR-16-1 [86]. In addition, with increasing the levels of miR-16, p53 targets an important survival protein called Survivin and also inhibits proliferation of colon tumor cells [87]. Some miRNAs also apply their tumorigenic effect by targeting p53, and some other miRNAs enhance the antitumor function of p53. For example, miR-19b with targeting p53 can increase tumor growth, inhibit apoptosis, and increase invasion and metastasis, so it is suggested to inhibit this miRNA by using appropriate antagomiR as an appropriate therapeutic approach [88].

In contrast, miR-766 can inhibit the cell cycle by enhancing the function of p53 [89]. In addition, some studies have shown that increasing the expression of miR-203 and miR-22 in colon cancer cells with a mutation in *TP53* can cause induction of apoptosis, inhibition of proliferation, decrease in survival and also increasing sensitivity to chemotherapy [90, 91] (Fig. 3C).

### Inflammation, DNA aberrant methylation and tumorigenesis: what are the miRNAs’ roles?

**Fig. 4:**
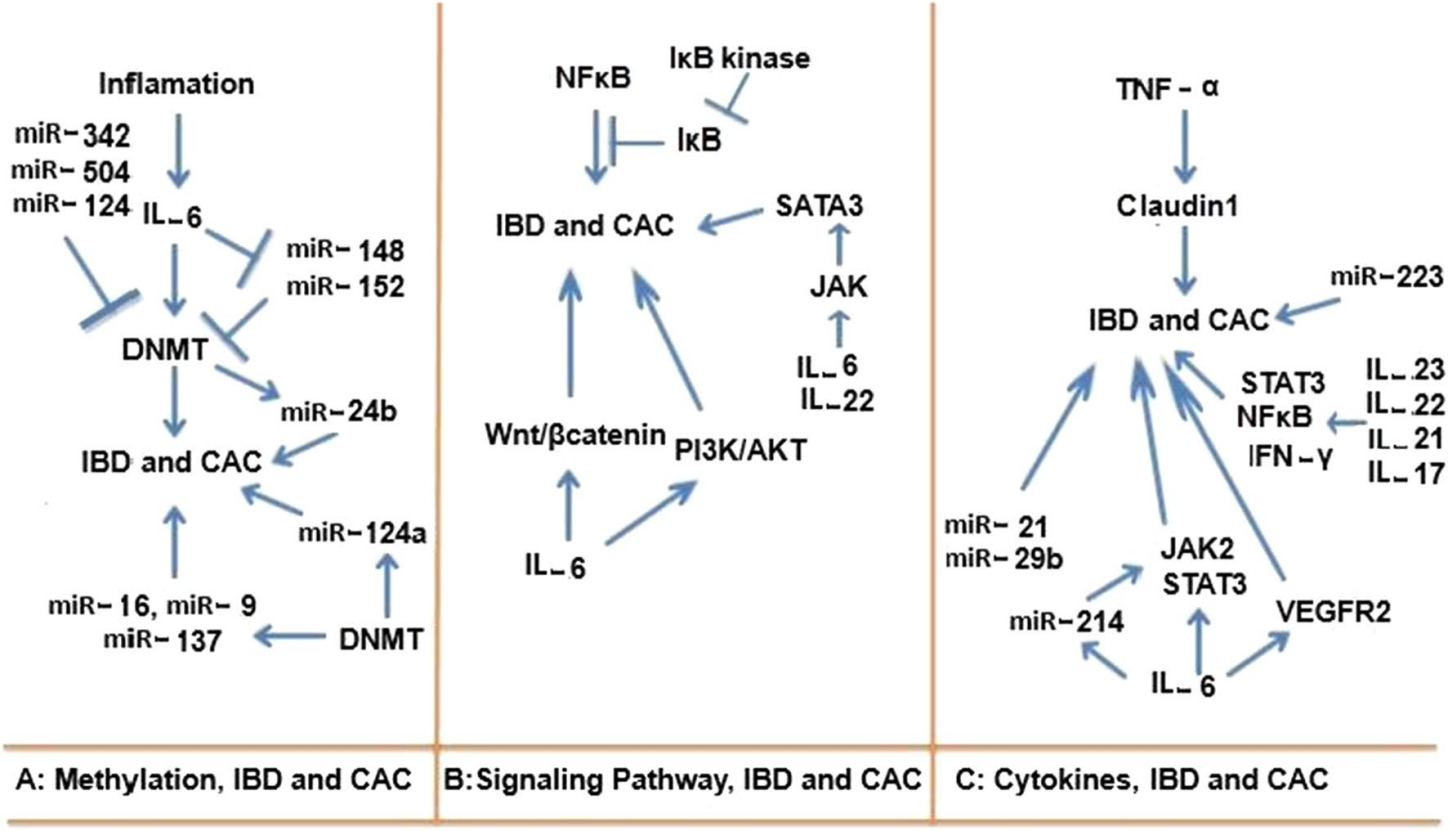
Crosstalk between inflammatory factors, miRNAs in Methylation, signaling pathways and cytokine functions during IBD and CAC

DNA methylation in CpG Island regions of promoter of genes is one of the ways to silence some genes and is a highly regulated process. Disruptions in this setting like hyper methylation of tumor suppressor genes can have catastrophic consequences. DNA methylation is performed by DNA methyl transferase enzymes (DNMT) and up to now three DNMT enzymes have been identified: DNMT1, DNMT3A and DNMT3B [92]. One of the destructive events that occur in IBD appears to be the increased expression of DNMT1 and DNMT3B in colon epithelial cells and hypermethylation of some important genes in inflammation and cancer such as some tumor suppressor genes, which is observed in active phase of disease [93, 94]. In pre-tumor cells as well as CAC tumor cells, the increased expression of DNMT1 has been reported compared to the sporadic type [95]. During chronic inflammation, cytokines such as IL-6 stimulate the expression of DNMT1 enzymes, and IL-6 possibly by inhibiting miR-148a and miR-152 plays an incite role because these miRNAs reduce the expression of DNMT1 enzyme [96, 97]. Hyper methylation of some miRNAs in colorectal tumor cells has been observed compared to healthy cells, which seem to play a role in weakening proliferation and inhibition of metastasis [98–101]. In addition, increased miR-124a methylation raises the risk of colorectal cancer development in IBD patients, and likely the level of this methylation is directly related to the duration of IBD [102]. Considering the significance of this finding, the evaluation of methylation level of some miRNAs including miR-124, miR-16, miR-9 and especially miR-137 as a prognostic marker has been suggested to identify high-risk individuals for colorectal cancer development among IBD patients [103]. Some miRNAs play an antitumor role with targeting DNMT enzymes. For example, miR-506 and miR-124 inhibiting colorectal cancer progression and increasing sensitivity to chemotherapy by targeting DNMT1 and DNMT3B [104]. miR-342 is involved in inhibiting of proliferation and invasion of colorectal tumor cells by targeting DNMT1 [105] (Table 1). miR-143 also plays its tumor suppressor role by targeting DNMT3A [106] (Fig. 4A).

### Signaling pathways, IBD and cancer; a brief overview

As previously mentioned, some of the signaling pathways such as NFκB, STAT3 and AKT/PI3K pathways play a very important role in the development and progression of CAC. NFκB is a transcription factor that has a great importance in the inflammation process. NFκB has two subunits called p50 and p65, which form a heterodimer complex. NFκB is in the cytoplasm and under the Inhibition of IKB in normal state is inactive for transcription. Pro-inflammatory cytokines and free radicals activate the enzyme called IKB kinase. This enzyme with IKB phosphorylation causes IKB ubiquitination and degradation. Subsequently, NFκB is released and goes to the cell's nucleus, where it increases the transcription of the genes involved in inflammatory response [107]. STAT3 is also a transcription factor involved in inflammation, cell growth and is also very important in controlling apoptosis. STAT3 is also present in cytoplasm and in response to inflammatory cytokines, especially IL-6 and IL-22 under the influence of JAK that is phosphorylated and comes in the form of dimer and goes to the nucleus [108, 109]. It seems that STAT3 has a twofold role in the pathophysiology of IBD. Though its important role in differentiating of Th17-effector has been specified, it affects Treg cells. Indeed, it affects the process of inflammation both stimulatory and inhibitory [110, 111]. The activation of STAT3 signaling pathway in IL-22-induced epithelial cells increases the cell survival and inhibits apoptosis, which is indicative of the importance of this signaling pathway in progressing IBD to cancer [112]. In addition to STAT3, NFκB signaling pathway is also very important in inhibition of apoptosis in epithelial cells, as some studies have shown that inactivation of NFκB pathway inhibits the expression of anti-apoptotic genes and reduces tumor size in animal models of CAC [113]. In human studies, an increase in the expression of NFκB in colon and peripheral blood has been also reported in patients with colorectal carcinoma [114, 115]. There is an interesting association between PI3K/AKT and NFκB signaling pathways. AKT/PI3K signaling pathway can also play an important role in the pathogenesis of CAC. This signaling pathway is started by the attachment of an extracellular ligand such as IL-6 to the cell surface receptors leading to phosphorylation and activation of PI3K. Active PI3K activates a secondary messenger called PIP3 with phosphorylation of membrane lipids, and this is PIP3 that activates AKT. AKT enhances cell survival, inhibits apoptosis and also increases cell proliferation [116, 117].

AKT can decompose IKB and release NFκB by phosphorylation of IKB kinase leading to enhancement of NFκB signaling [118]. Additionally, AKT is also associated with WNT/β-Catenin signaling pathway. Some studies have shown that activation of WNT/β-Catenin signaling pathway in T-effector cells, especially Th17 and Treg cells is involved in development of CAC [119, 120]. The above-mentioned signals are the most important signaling pathways involved in CAC development and progression. However, transcription factors and other signaling pathways are also related to CAC, among which SMAD proteins and aryl hydrocarbon receptors can be mentioned [121, 122] (Fig. 4B).

### Cytokines, signaling pathways and CAC: what’s the role of miRNAs?

The activation of the NFκB and STAT3 signaling pathways in intestinal epithelial cells plays a major role in resistance to apoptosis and tumorigenesis. Some cytokines produced by immune cells have a receptor on the epithelial cells of intestine and can activate these signaling pathways in epithelial cells and enhance tumorigenesis. Therefore, one of the reasons for high risk of colorectal cancer in IBD patients is continuous production of these cytokines due to chronic inflammation. In this section, we review the most important role of these cytokines in the progression of chronic bowel inflammation to colorectal cancer, and discuss the association of miRNAs with these cytokines and their dependent signaling pathways.

#### TNF-α

One of the key cytokines in the pathogenesis of IBD and CAC is TNF-α. As previously mentioned, in IBD, due to mucosal barrier dysfunction and penetration of bacteria into lamina propria, macrophages, neutrophils and NK cells are also emitted to this region. Neutrophils and macrophages release high levels of TNF-α, and it seems that this cytokine plays an essential role in the continuation of inflammatory responses. TNF-α, in addition to further damaging of mucosal barrier of intestine, can enhance the recruitment of other immune cells to lamina propria and stimulate the production of other inflammatory cytokines by attaching to its receptors on the epithelial cell surface as well as pre-tumor cells. TNF-α enhances NFκB signaling and, subsequently, stimulates inflammation, tumorigenesis, and proliferation, and increases the survival of pre-tumor cells [13, 113, 123].

TNF-α role in tumorigenesis is in interaction with claudin1. Some studies have suggested the role of claudin1 in development of CAC, also cladin1 inhibition decreases TNF-α effects on colon tumor cell proliferation and invasion [124–126]. Targeting TNF-α is a therapeutic approach in the treatment of IBD. Attenuation of TNF-α effect by inhibiting claudin1 can be studied as a therapeutic approach to inhibiting CAC, as well as preventing progression of IBD to cancer. Some miRNAs may also be useful therapeutic targets in this regard. It seems that miR-155 enhances the invasion and migration of tumor cells, through increasing the expression of claudin1 [127] (Table 1). This miRNA also plays an active role in pathogenesis of IBD and it is probably one of the miRNAs that contribute to the destruction of tight junction and weakening of intestinal mucosal barrier [13, 128]. Therefore, miR-155 can be considered as a therapeutic goal both in IBD and CAC. Although the effectiveness of inhibiting this miRNA has been studied in treatment of certain cancers like nasopharyngeal cancer and it has been shown that inhibition of this miRNA inhibits the migration of tumor cells. It seems that the therapeutic efficacy of miR-155 inhibition in CAC has not yet been studied [129]. miR-19a can also be one of the therapeutic targets in CAC. TNF-α exerts its stimulatory effect on NFκB signaling pathway and the development of CAC by increasing the expression of miR-19a [130]. Another miRNA that appears to be related to TNF-α is miR-105. It has recently shown that TNF-α stimulates the expression of miR-105 and this miRNA stimulates the invasion and metastasis of colorectal cancer cells [131]. There is no doubt that further studies will reveal more about the association of miRNAs with TNF-α in IBD and CAC.

#### IL-6

IL-6 is key cytokine in the pathogenesis of IBD and CAC. IL-6 is mainly released from the inflammatory macrophages and neutrophils and T cells. It can activate all the signaling pathways described above [132]. NFκB plays a major role in production of IL-6 in immune cells, and IL-6 has effects on intestinal epithelial cells through its receptors and activates JAK2-STAT3 signaling pathway [132, 133]. IL-6 strengthens the differentiation of Th17 cells and somehow plays a role in production of IL-17 as an important inflammatory and tumorigenic cytokine [134]. Some studies have shown that IL-6 is involved in increasing the proliferation of tumor cells in colon and NFκB/IL-6/STAT3 signaling seems to play a crucial role in CAC development [133]. In addition, it seems that IL-6 strengthens angiogenesis by stimulating the expression of vascular endothelial growth factor receptor 2 (VEGFR2) on the surface of tumor cells. Therefore, inhibition of IL-6 receptor with monoclonal antibodies has been suggested as a therapeutic approach to treatment of colorectal cancer [135, 136]. There is an interesting relationship between some miRNAs and IL-6. Expression of miR-21 and miR-29b is stimulated in colon tumor cells in the presence of IL-6. MiR-21 is known as an oncogenic miRNA. This miRNA increases proliferation, inhibits apoptosis and even increases the ability of metastases in tumor cells probably by inhibiting PTEN expression, which is an inhibitor of AKT/PI3K signaling pathway. More interestingly, the secretion of miR-21 and miR-29b by tumor cells has effect on immune cells and increases the production of IL-6 [137]. In miR-21 knock out mouse models, reduced expression of IL-6, IL-23, IL-17, IL-21 and STAT3 have also been observed. In addition, the size and number of tumor cells has also been decreased in these mice. MiR-21, as well as miR-224 and miR-452 stimulate the proliferation of tumor cells by stimulating WNT/β-Catenin signaling pathway. MiR-21 is also involved in activation of NFκB by targeting PDCD4 as an important tumor suppressor protein and NFκB inhibitor [138–141]. Targeting oncomiRs such as miR-21 and miR-181-b, and lack of PDCD4 in colitis and CAC mice models have been associated with enhanced IL-6 production, intensified activity of STAT3 signaling pathway, enhanced colitis and proliferation of tumor cells [142, 143].

Considering these findings, it seems that, miR-21 may be an important player in development and progression of CAC. More importantly, inhibition of the expression of this miRNA using LNA-antimiR-21 has been proposed as a therapeutic approach to the treatment of colorectal adenocarcinoma [144, 145]. The role of miR-21 in the pathogenesis of IBD is also very interesting. Some studies have shown that this miRNA is involved in the destruction of mucosal barrier of intestine as the most important events in developing chronic inflammation and pathogenesis of IBD. In addition, it appears that miR-21 intensifies the inflammatory process by increasing the expression of IL-6, macrophage Inflammatory Protein 2 (MIP2) and TNF-α [146, 147]. Therefore, miR-21 along with cytokines such as IL-6 is may be a very important link between IBD and cancer. MiR-34a is another miRNA linked to IL-6, however, its effects on IL-6 and tumorigenesis is inhibitory. By targeting IL-6 receptor (S-IL-6-R1), this miRNA inhibits the effect of IL-6 on epithelial cells and attenuate IL-6/STAT3 signaling and thereby has an inhibitory effect on tumorigenesis, invasion and metastasis. The expression of this miRNA can be enhanced by the p53, a well-known tumor suppressor [148, 149]. It seems that, IL-6 can activate STAT3 signaling by stimulating miR-214 expression in colon tissue. MiR-214 can also play a role in tumorigenesis beside its role in IBD pathogenesis. This miRNA stimulates NFκB signaling pathway, and strengthens AKT/PI3K signaling pathway by reducing PTEN expression. Furthermore, this miRNA increases the disease severity in IBD patients and stimulates progresses towards CAC. Some studies have shown that the inhibition of this miRNA reduces: (1) the number and size of tumors in mouse models, (2) the proliferation of colon tumor cells, and (3) the severity of inflammation in colon tissue of Ulcerative colitis patients [13, 150–152]. Another miRNA that has an anti-inflammatory and anti-tumor role is miR-139-5p. This miRNA can reduce the expression of IL-6 and TNF-α in colorectal cancer cells, possibly by suppressing NFκB signaling [153]. This miRNA has an inhibitory effect on NFκB and STAT3signaling pathways. One study have shown that the activity of STAT3, NFκB, and MAPK signaling pathways have been enhanced, and more severe inflammation and tumorigenicity can be occurred in on miR-139-5p knock out mice [154]. Another miRNA with anti-inflammatory properties is miR-200b that is also associated with IL-6/STAT3 signaling, TNF-α and NFκB. This miRNA not only plays a role in enhancing the mucosal barrier of intestine, it also weakens the tumorigenesis process in mice models of colitis. In addition, this miRNA seems to play an important role in inhibiting metastasis and increasing chemotherapy sensitivity in patients with colorectal cancer [123, 155–157] (Table 1).

The role of miR-200b in reducing the resistance to chemotherapy is very interesting and it seems that some other miRNAs such as miR-146a in an antagonistic action increase drug resistance in colorectal cancer cells [158]. These new findings promise that using RNA mimics and antagomiR in the future may overcome the drug resistance in cancers.

#### IL-23

In addition to IL-6 and TNF-α, other cytokines such as IL-22 and IL-17 can directly affect epithelial cells. Th17 cells that play a very active role in the pathogenesis of IBD and in addition to IL-17, secrete other cytokines including IL-21, IL-22, and IL-6 [13, 57, 159]. The production of cytokines by Th17 cells is largely dependent on IL-23. In IBD innate immune cells such as macrophages that penetrated the lamina propria secrete high levels of IL-23. This cytokine causes the differentiation and recruitment of Th17 cells and by binding to its receptors on the surface of these cells activates STAT3 signaling pathway and subsequently produces IL-17 and IL-22 [160–162]. These cytokines can exert their tumorigenic effects by effect on epithelial cells and activating NFκB and STAT3 signaling pathways [161, 163]. miRNAs are related to all the above-mentioned events and have effect on the differentiation, cytokine producing ability of T cells [13]. For example, miR-223 is associated with IL-23, and it seems that IL-23 (through this miRNA) can attenuate the production of a key tight junction protein called Claudin-8, and apply its damaging effect on mucosal barrier of intestine through this process which is resulted in a greater penetration of bacteria into lamina propria that is led to the penetration of immune cells and continuation of inflammatory reaction [164, 165]. It seems that, miR-29 has an inhibitory effect on the secretion of IL-23 in patients with IBD [166]. According to the above results, it seems that IL-23, in addition to its role in the pathogenesis of IBD and its destructive effects on intestinal mucosal barrier, which can lead to chronic intestinal inflammation, is able to activate STAT3 signaling and enhance the production cytokines such as IL-17 and IL-22. These cytokines can also affect colon epithelial cells and enhance tumorigenesis, which will be discussed below. Therefore, inhibition of miRNAs such as miR-223 and enhancement of miR-29 expression can be useful in attenuating the destructive effects of IL-23 and have an inhibitory effect on the progression of chronic intestinal inflammation to colorectal cancer.

#### IL-17

IL-17 expression appears to be increased in the colon tissue of IBD patients (both Crohn’s disease and Ulcerative colitis) and some studies have also shown that IL-17 plays a key role in the pathogenesis of colitis in mice[167, 168]. It is apparent from studies on mouse models that IL-17 may play a very important role in development of CAC. One study of mouse models of CAC has clearly shown that IL-17 deficiency in mice can reduce p-STAT3 and p-AKT levels and attenuate tumorigenesis. [169], suggesting that IL-17 may enhance the progression of chronic bowel inflammation to colorectal cancer by amplifying STAT3 and PI3K/ AKT signaling. One study also showed that inhibition of IL-17 by antibodies had positive effects in attenuating the progression of colitis to colorectal cancer in mice [170]. Therefore, miRNAs such as miR-146a that appear to stimulate the production of this cytokine can be further studied as therapeutic target [171]. Some other miRNAs including miR-193a-3p and miR-23-b also inhibit the function of IL-17 cells, as it has been shown that miR-193a-5p weakens the production of one type of IL-17 receptor called IL-17RD. More interestingly, the expression of this miRNA is reduced in CAC [172, 173]. Therefore, the use of miR-223 mimic and miR-193a-5p mimic may have positive effects in weakening the progression of chronic intestinal inflammation to colorectal cancer. It appears that miR-124 can also reduce IL-17 expression in Th17 cells by reducing STAT3 expression and attenuating its binding activity to the IL-17 promoter. miR-124 mimic also had positive effects on attenuating CAC development in mice [174]. Some studies have shown that reducing the level of this miRNA in intestinal tissue of pediatric patients with Ulcerative colitis increases the expression and activity of STAT3, subsequently exacerbates inflammation [175] (Table 1). Increasing the methylation of the gene of miR-124a is also associated with the risk of CAC in patients with Ulcerative colitis [102]. Therefore, miR-124 can be further studied as one of the therapeutic targets for weakening the progression of chronic intestinal inflammation to colorectal cancer.

#### IL-21

In addition to increasing the differentiation of Th17 cells, IL-21 also appear to attenuate the production of IFN-γ by Th1 cells which may lead to attenuation of CD8^+^ T cells function in targeting tumor cells. Therefore, it is not surprising that lack of IL-21 reduces the severity of inflammation and tumorigenesis in mouse models [176–178]. Although IL-21 appears to be involved in enhancing the Th2 response, significant expression of this cytokine in intestinal tissue has been reported in both patients with active Ulcerative colitis and patients with Crohn's disease [177, 179, 180]. A study of sporadic colorectal cancer has shown that IL-21 deficiency may be associated with impaired STAT3 and NFκB signaling activity in immune and neoplastic cells [181]. However, the association of IL-21 with these signaling pathways in the CAC is not yet clear. In addition, the relationship between IL-21 and miRNAs has not yet been well studied. It seems that miR-30a can target the receptor of this cytokine and inhibit the differentiation of Th17 cells [182]. The stimulatory effect of miR-155 on the production of IL-21 is also reported in a study conducted on patients with lupus erythematosus [183]. Previously, the role of miR-155 in the pathogenesis of IBD and colorectal cancer were mentioned, therefor, clarifying the association of this miRNA with IL-21 in CAC may be helpful.

#### IL-22

IL-22 is also one of the most important cytokines in development and progression of CAC. IL-23, IL-21 and IL-6 play a role in production of IL-22 through the STAT3 signaling. IL-22 can also stimulate its production by activating STAT3 signaling pathway. IL-22 is another cytokine that directly affect intestinal epithelial cells and plays an important role in wound healing. This cytokine can also be secreted by Th1, Th22, NKp44 + ILC3s cells and even NKT cells in addition to Th17 cells [40, 184]. IL-22 not only activates STAT3 signaling pathway in epithelial cells and involved in tumorigenesis and CACs, it also appears to be involved in the resistance to chemotherapy in colon cancer cells [185]. IL-22 is regulated by aryl hydrocarbon receptor signaling pathway [184]. This receptor plays a role in attenuating the function of Th17 cells, attenuating inflammation, and wound healing [13, 186, 187], In addition, this receptor may attenuate tumorigenesis in mouse models of colitis by increasing the expression of miR-132 [188].

It has also shown that inhibiting the expression of this receptor by miR-124 exacerbates inflammation in IBD [189]. However, aryl hydrocarbon receptor probably stimulates the production of IL-22 by affecting Th22 cells [184, 190], mighty due to the role of this receptor and IL-22 in wound healing. MiR-155 is also associated with IL-22. This miRNA stimulates the production of IL-22 by Th17 cells, which applies this effect by targeting jarid2 as a DNA-binding protein with the inhibitory properties on cytokines production [184]. Though jarid2 has inhibitory effects on differentiation of Th17 cells and inflammation process, it has a stimulatory effect on EMT which is shown in lung cancer cells, bladder, and clone while its inhibition is suggested as a therapeutic approach in treatment of bladder cancer [191–194]. Therefore, it seems that miR-155 cannot be regarded as merely oncogenic and many dimensions of the functions this miRNA, jarid2 and IL-22, their association with one another as well as their association with other miRNAs such as miR-219-5p, a miRNA with inhibitory effect on EMT, are still unclear [195, 196].

#### IL-10

The role of Treg cells in the pathogenesis of IBD and CAC is very important. As previously mentioned, these cells play a role in regulating the function of T-cells and immune tolerance. Anti-inflammatory function of these cells is associated with a transcription factor called FOXP3, but PORγ expression including anti-inflammatory and colitis preventative properties, along with FOXP3 seem to increase Immunosuppressive properties of these cells [197, 198]. Treg cells play a role in preventing the development of CAC, although the presence of a tumor suppressor protein in epithelial cells (called RUNX3) is necessary to enhance the action of Treg cells in preventing the colitis progression toward cancer [199–201]. miR-532-5p by targeting this protein plays a stimulatory role in development and progression of colorectal cancer[202]. Treg cells secrete IL-10 and TGF-β. In addition to Treg cells, IL-10 is also secreted by macrophages and is among cytokines that play an important role in IBD and CAC [13, 203, 204]. Some studies have shown that the level of IL-10 in patients with colorectal cancer is increased, and a positive correlation is found between the level of this cytokine and the proliferation of colon tumor cells [205, 206]. In addition, IL-10 has been suggested as a prognostic marker of recurrence after the treatment of colorectal cancer and seems to be associated with poor prognosis [207, 208]. However, IL-10 level is decreased in colon mouse models of colitis and seems to play a protective role against the IBD [209, 210].

Therefore, the role of this cytokine in CAC is probably in the late stages and due to its immunosuppressive properties. Accordingly, macrophages have effect on colon tumor cells by IL-6 production and by activating STAT3 signaling pathway stimulate the production of IL-10. This cytokine by weakening the function of CD8^+^ killer cells promotes the progression of tumors and resistance to chemotherapy [203, 208]. The association of miRNAs with IL-10 in CAC is not yet clear. Recently, it has also been shown that by using a long non-coding RNA (called GAS5) and inhibiting IL-10 and VEGEF-A, progression of colorectal cancer can be inhibited. In addition, GAS5 induces apoptosis and inhibits the proliferation of colorectal tumor cells by applying inhibitory effects on miR-182-5p and miR-221 [211–213]. miR-221 and miR-222 appear to have a tumorigenic effect and can activate STAT3 signaling pathway. One study on mice models of colitis showed that the inhibition of these miRNAs can be associated with reduced tumorigenesis [214]. Therefore, investigating the relationship between these miRNAs and IL-10 in CAC can be an interesting topic for future studies. A study of mouse models showed that knocking down miR-106-a could weaken ileal inflammation. The results of this study also showed that a deficiency of this miRNA could enhance the suppressive function of Treg cells and increase IL-10 production [215] (Table 1). These findings may indicate the destructive role of miR-106-a in chronic intestinal inflammation, but the effects of the association of this miRNA and IL-10 on the progression of chronic intestinal inflammation to colorectal cancer are unclear. MiR-98 appears to be a negative regulator of IL-10 production in macrophages [216]. It appears that this miRNA can attenuate the proliferation of colorectal cancer cells and enhance apoptosis [217]. Therefore, examining the relationship between this miRNA and IL-10 in CAC could be an interesting topic for future studies and may be useful in elucidating the mechanism of IBD progression to colorectal cancer.

#### TGF-β

TGF-β is another cytokine secreted by Treg cells. This cytokine has a dual role in the inflammation process. TGF-β can stimulate the differentiation of Th17 cells. On the other hand, this cytokine can attenuate the production of inflammatory cytokines in macrophages, by inhibiting NFκB. In addition, TGF-β has a stimulatory effect on the differentiation of Treg cells [218–220]. TGF-β mainly transmits its message to the cell through transcription factors called SAMADs [221]. Although TGF-β has anti-inflammatory effects and can exert an inhibitory effect on the proliferation of epithelial cell, and may play a role in preventing the progression of IBD to colorectal cancer, it seems that this cytokine also has a stimulatory role in the late stages of tumorigenesis. Few studies have examined the association between miRNAs with TGF-β signaling in the CAC, and more studies are needed. Studies have shown that some miRNAs such as miR-27a and miR-140-5p can exert their tumor suppressant effects on colorectal cancer cells by targeting SMAD2 as a key component of TGF-β signaling [222–225] (Table.1). MiR-155 may also have an inhibitory effect on TGF-β/SMAD signaling. In a study performed on mouse models of CAC, it was shown that the activity of this signaling pathway is enhanced in miR-155^−/−^ mice, and this increase in activity is associated with enhanced tumorigenesis [226].

## Conclusion

Chronic inflammation, which is the main characteristic of IBD, increases the amount of free radicals and also some inflammatory cytokines such as TNF-α, causing damage to the DNA of colon and rectum epithelial cells. Over time, with the function of some cytokines that are continuously produced due to the chronic inflammatory response and their associated signaling pathways, the cell which is susceptible to be cancerous, survives apoptosis and goes from Dysplasia to colorectal carcinoma. Some miRNAs are associated with these events and have been studied as therapeutic targets. These miRNAs are important actors in this dangerous pathway, requiring more attention in future.

## Data Availability

Data sharing is not applicable to this article as no new data were created or analyzed in this study.
